# Early Results of a Variable-Angle Volar Locking Plate for Distal Radius Fractures: A Bi-centre Study

**DOI:** 10.7759/cureus.18321

**Published:** 2021-09-27

**Authors:** Sherif Elerian, Talvinder Singh, Nikolas A Jagodzinsk, Rory Norris, Simon Tan, Dominic Power, Jonathan Jones, Zehong Chen, Hassan Eltagy

**Affiliations:** 1 Trauma and Orthopaedics, Sandwell General Hospital, Birmingham, GBR; 2 Hand Unit, University Hospitals Birmingham NHS Trust, Birmingham, GBR; 3 Trauma and Orthopaedics, Peterborough District Hospital, Cambridgeshire, GBR

**Keywords:** distal radius fracture, variable-angle locking plate, cam grip, joystick, early outcomes

## Abstract

Purpose

This study examines the clinical, functional and radiological outcomes of distal radius fracture fixation with the Aptus® (Medartis, Pennsylvania) locking plate in order to determine its efficacy and identify notable findings related to treatment variations.

Methods

This is a retrospective bi-centre study collecting patient details from a district general hospital and a regional hand unit. We assessed 61 consecutive patients with distal radius fractures (Arbeitsgemeinschaft für Osteosynthesefragen (AO) grade A=19, B=9, C=33) fixed using an Aptus® plate with a minimum of six months follow-up. Outcome measures included the DASH (Disabilities of the Arm, Shoulder and Hand) score, wrist range of movement and grip strength, and complications. Radiographs were reviewed to assess restoration of anatomy and union.

Results

All but two fractures united within six weeks. The mean ranges of movement were only mildly restricted compared to the normal wrist (flexion/extension = 102°; radial/ulna deviation = 53°; pronation/supination = 169°). Mean postoperative grip strength was 23.8 kg, which was comparable to the contralateral side at 31.5 kg. The mean DASH score was 18.2. Seven patients had screws misplaced outside the distal radius although three of these remained asymptomatic.

Conclusion

Variable-angle locking systems benefit from the flexibility of implant positioning and may allow enhanced inter-fragmentary reduction for accurate fixation of intra-articular fractures.

## Introduction

Fractures of the distal radius are common injuries and occur in patients of all ages [1-2]. The ageing population is causing a rapidly increased incidence of fragility fractures of the distal radius [3]. Anatomically designed volar locking plates have seen a surge in use over the last decade with mainly good functional and radiological results being reported [4-6]. Despite the plate not providing a dorsal buttress, the angular stability afforded is mechanically strong enough to hold the reduction of dorsally comminuted and dorsally tilted fractures in fragile bone [7-8]. Stability is not dependent upon friction between the plate and bone so the periosteal blood supply is maintained [8]. Locking plates provide cantilever loading, preventing forces running through the fracture site by bridging the fracture giving comminuted and osteoporotic fractures a better chance to heal.

Plate designs are now more ‘anatomical’ and low-profile with distally projecting screws or pegs at varying, fixed angles that provide a raft under the curves of the distal articular surface of the radius. The ability to keep the plate behind the ‘watershed line’ reduces the incidence of tendon irritation and the anatomical design of the plate aids fracture reduction [2,5,9-12].

More recent designs of volar locking plates allow for variable angles of the trajectory of the locking screws (poly-axial screws). The shape of the curve of the distal radius may vary considerably between patients and variable-angle plates may allow for a more customised supporting raft of subchondral screws [8]. Poly-axial screws may also be better for independently addressing each fracture fragment in intra-articular fractures [2,12]. We explore whether or not they are an improvement or add unnecessary complexity over fixed-angle devices.

We have performed a bi-centre, retrospective study comparing all patients with fractures of the distal radius fixed with Aptus® (Medartis, Pennsylvania) variable-angle volar locking plates at a regional hand unit (RHU) and a district general hospital (DGH). We have compared functional, radiological and patient-rated outcomes with a mean follow-up of 12 months in order to determine its efficacy and to identify notable findings related to treatment variations.

## Materials and methods

Patients and methods

We have retrospectively collected data over a one-year period from all patients treated with the Aptus® plate from both centres, with a minimum of six months follow-up for each patient. Inpatient and outpatient notes were analysed for the mechanism of injury, any associated injuries, operation details, and the occurrence of any postoperative complications.

After discussion with the hospital ethics committees, it was ascertained that this trial was, in effect, an audit of existing practice in our units and formal approval was granted. The study was performed in accordance with the ethical standards of the 1964 Declaration of Helsinki as revised in 2000, and all patients gave informed consent prior to being included in the study. All operations were performed using a volar approach through the bed of the flexor carpi radialis tendon (FCR). All wrists operated on at a regional hand unit (RHU) were mobilised, free from casts and splints, by specialist hand therapists within seven days of surgery. At a district general hospital (DGH), the use of casts, splints, and therapy varied considerably depending on the surgeon’s preference.

At the patients’ final outpatient appointment, measurements of wrist ranges of movement were recorded with a standard goniometer, including flexion, extension, radial and ulnar deviation, and forearm pronation and supination. Grip strengths in kilograms (Kg) were compared with their unaffected side using a Jamar® level-2 dynamometer (Performance Health, Warrenville, IL) at a standardised grip length setting, taking the highest results from three rapid-exchange measurements. Their current pain scores were obtained using a scale between 10 = unbearable pain and 0 = no pain at all. Functional outcomes were assessed using the extended, three-module questionnaire version of the DASH patient-rated scoring system where a score of 100 = maximum disability and 0 = no disability.

Preoperative, intraoperative, and postoperative radiographs were reviewed for each patient. Fractures were classified using the Mϋller-AO (Arbeitsgemeinschaft für Osteosynthesefragen) comprehensive classification system and any articular step-off was recorded. The anteroposterior views were used to calculate radial height and radial inclination, and dorsal or volar tilt was calculated from the lateral view. We have compared our findings with that of Wong who defined a good reduction as postoperative films showing an articular step-off of <2 mm, a restoration of volar tilt past neutral, radial height >8 mm, and radial inclination >15˚ [8]. X-rays were taken on at least one occasion over six weeks postoperatively to assess bone healing but only if clinically indicated thereafter. X-rays were not repeated for the purpose of this study.

## Results

Sixty-one Aptus plates were used on 61 patients in 22 months at the two centres (Table 1). As the demographic data were similar between the two centres, the patients were considered as a single cohort although comparisons have been drawn where there is a difference in treatment rather than a difference in location. Demographics were recorded along with hand dominance, delay to surgery, and duration of postoperative follow-up. No patients were excluded from the consecutive series. Nine patients had incomplete follow-up. Four were excluded from the study, as they were lost to follow-up. However, five were followed up to the point of fracture union so their radiographic outcomes were included.

**Table 1:** Demographic data * RHU: Regional hand unit; DGH: District general hospital; #: Fracture; AO: Arbeitsgemeinschaft für Osteosynthesefragen

Demographic	RHU*	DGH*	Combined
No. Aptus plates used: Total (♂;♀)	28 (10♂; 18♀)	33 (10♂; 23♀)	61 (20♂; 41♀)
Age: Mean (range)	55.2 (22 – 89)	56.4 (24 - 88)	55.9 (22 – 89)
Dominant #; Non-Dominant # *	15; 13	13; 20	28; 33
AO Grade A	9	10	19
AO Grade B	4	5	9
AO Grade C	15	18	33
Consultant Surgeon; Registrars	28; 0	22; 11	50; 11
Delay to surgery: Mean (range)	11.1 days (2 – 26)	5.1 days (1 – 28)	7.7 (1 – 28)
No. Pts with incomplete follow-up six months post-op	3 (0♂; 3♀)	6 (3♂; 3♀)	9 (3♂; 6♀)
Mean duration of complete follow-up	10.9 months (6 – 21)	12.9 months (6 – 28)	11.9 months (6 – 28)

Mean (+/- SD (standard deviation)) pain score at final follow-up was 1.6 (+/- 1.8). Mean DASH score was 16.1 (+/- 17.5) (where 0 = no disability and 100 = maximum disability). Mean grip strength on the fractured wrist was 23.8 Kg (+/- 17.3), which was comparable to the mean grip strength on the unfractured wrist of 31.5Kg (+/- 18.7). Initial AO fracture classification did not correlate with either the DASH score (p=0.965), grip strength (p=0.209) or ranges of movement (Table 2) at final follow-up.

**Table 2:** Means and ranges of motion (in degrees) SD: Standard deviation

AO Fracture Classification	Overall	A	B	C	^*^p=
Mean Arc of Flexion / Extension (+/- SD)	102 (30)	107 (37)	102 (21)	99 (29)	0.194
Mean Arc of Lateral Deviation (+/- SD)	53 (18)	47 (19)	54 (10)	56 (19)	0.422
Mean Arc of Forearm Rotation (+/- SD)	169 (24)	165 (26)	177 (18)	168 (24)	0.236

There was no correlation between delay to operation from injury and DASH score (mean = 6 days; SD=7; p=0.66). Seven patients from the district general hospital (DGH) were operated on by specialist trainees as opposed to consultants. There was no correlation between the grade of the surgeon and the DASH score. The duration of postoperative casting was chosen as per the usual practice for each surgeon and not based on the complexity of the fracture (Table 3). Using the Mann-Whitney test to correlate arcs of motion with the duration of postoperative casting in weekly increments, the most statistically significant cut-off before wrists became stiff was four weeks for forearm rotation and lateral deviation (p=0.019 and p=0.008, respectively). There was no statistically significant cut-off (Table 3) for the arc of flexion-extension (p=0.690).

**Table 3:** Means and ranges of motion against the duration of postoperative immobilization (in degrees) * No. Patients: Number of patients; SD: Standard deviation

Duration in cast	< 1 week (No. Patients = 26) *	1 – 3 weeks (No. Patients = 11)	≥4 weeks (No. Patients = 15)
Mean Arc of Flexion-Extension (+/- SD)*	104.9 (29)	122.5 (24)	98.1 (27)
Mean Arc of Radial-Ulnar Deviation (+/- SD)	58.9 (15)	60.6 (7)	42.5 (20)
Mean Arc of Forearm Rotation (+/- SD)	176.7 (10)	178.8 (11)	156.7 (35)

**Figure 1:**
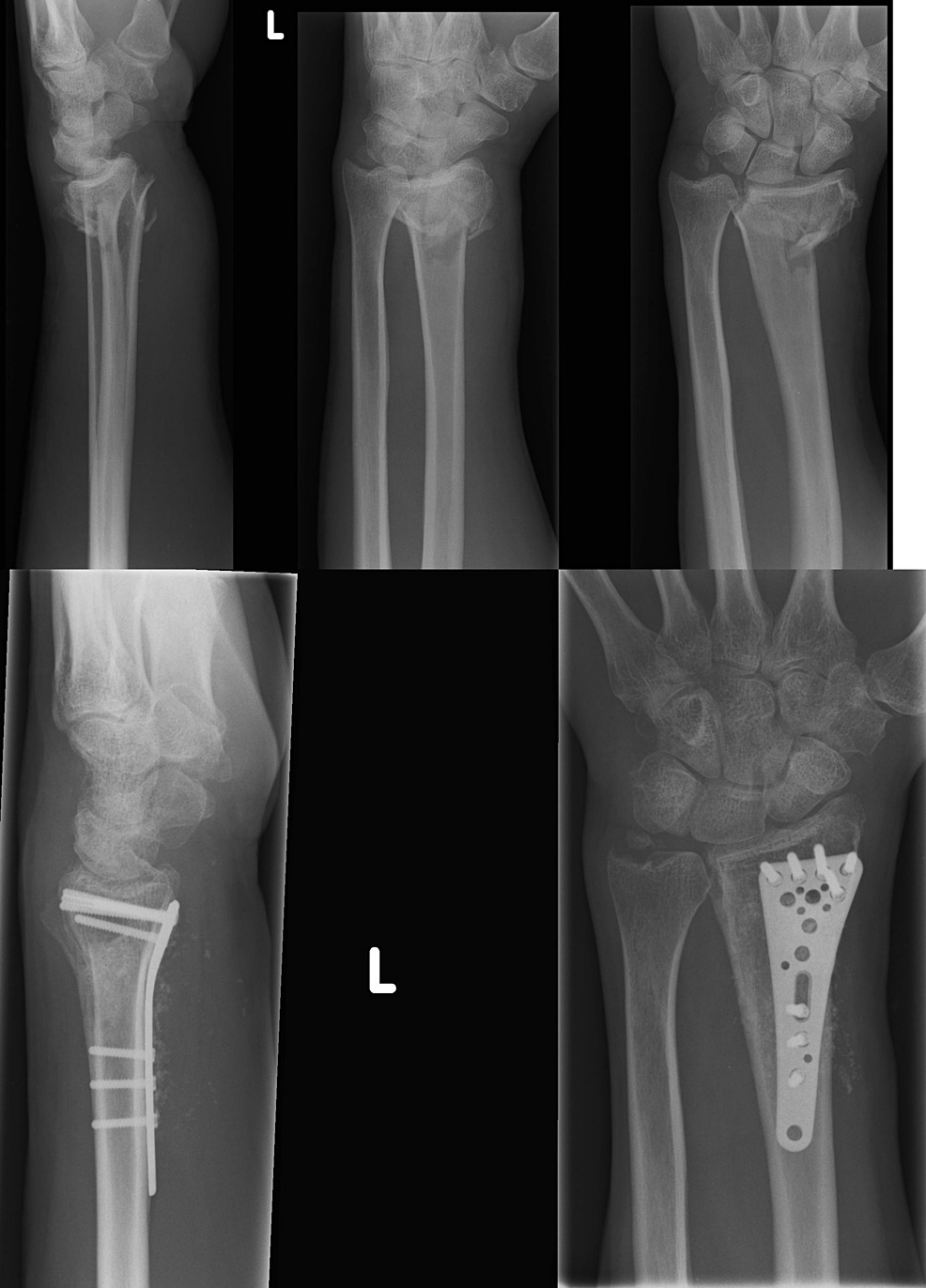
Pre and postoperative radiographs of an Aptus® trauma plate with a good raft of screws placed under subchondral bone and the AO 23-B1 fracture well reduced AO: Arbeitsgemeinschaft für Osteosynthesefragen

The positions of all fractures were improved with surgery. Wong defined a good reduction as postoperative films showing an articular step-off of <2 mm, a restoration of volar tilt past neutral, radial height >8 mm, and radial inclination >15˚ [8]. All of these parameters were achieved in 31 wrists (54%) in our cohort but this made no difference to clinical outcomes (Figure 1).

All fractures demonstrated good reduction based on Wong's criteria on radiographs taken six weeks postoperatively. All but two reductions were maintained until fracture union. Thirty-eight patients (62%) sustained associated distal ulna fractures of which five had operative fixation. All of these patients were treated by specialist hand consultants at the Hand Surgery Centre. Two were fixed with tension-band wiring, one with a plate, one with a screw and one with soft tissue bone anchors. One of these patients was lost to follow-up and, as the numbers of patients in this group are so small, it is difficult to draw any meaningful conclusions. Two patients developed carpal tunnel syndrome several months postoperatively and both were treated successfully by the decompression and removal of metalwork (Table 4). One of these patients also underwent a tenosynovectomy and the other was noted to have an injury to the cutaneous branch of the median nerve. This was probably caused during the initial approach, which, on this occasion, was ulnar to flexor carpi-radialis tendon (FCR).

**Table 4:** Complication rates

Complication	No. Patients
Total	11 (18%)
Minor	7 (11%)
Superficial wound infection	1
Carpal tunnel syndrome	2
Superficial nerve damage	1
Tenosynovitis	1
Asymptomatic screw misplacement	3
Major	4 (7%)
Symptomatic screw misplacement	4
Tendon rupture	0
Deep wound infection	0

**Figure 2:**
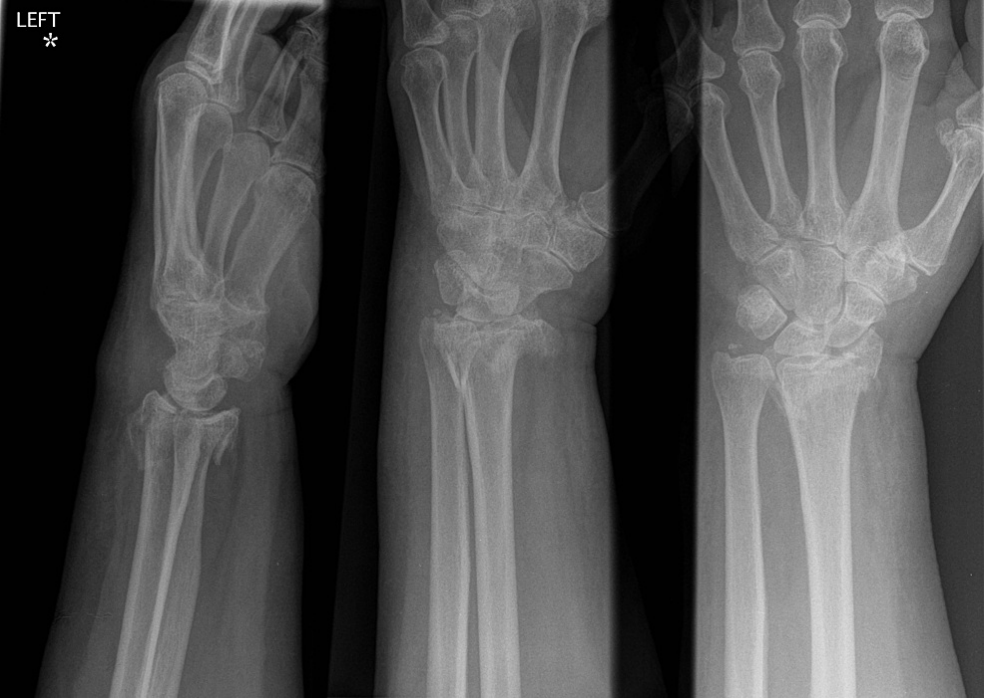
Preoperative radiographs of a patient whose fracture was fixed with the Aptus® osteotomy plate

**Figure 3:**
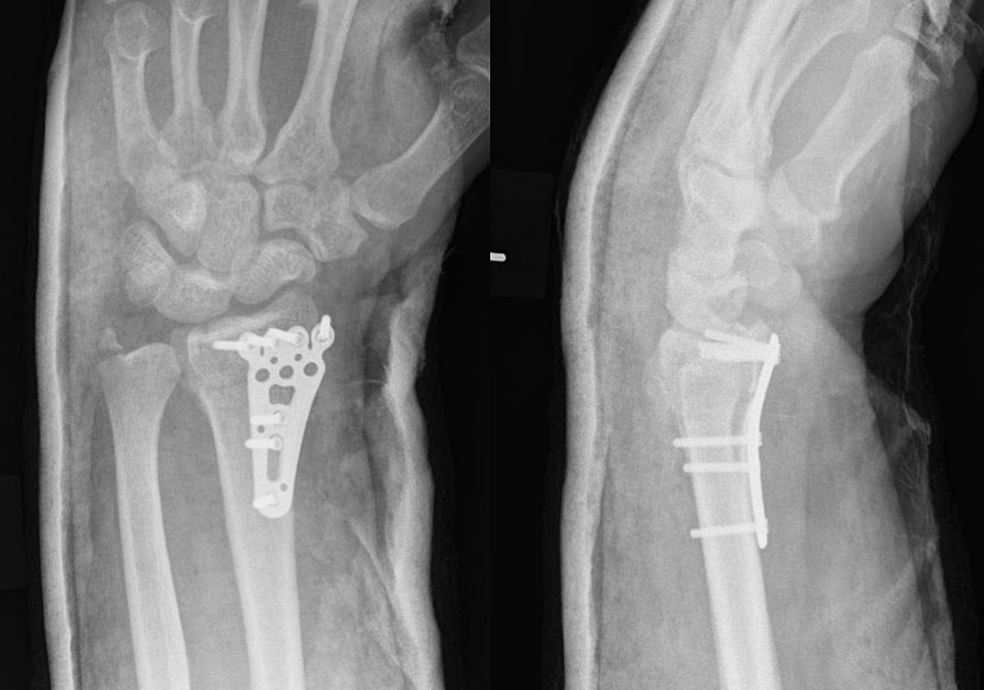
Postoperative radiographs showing a misplaced screw radial to the radial styloid and locking screws too short for osteoporotic bone

Seven patients had distal locking screws misplaced. All of these fractures were AO grade C and were technically difficult reductions (Figure 2). One was placed into the distal radioulnar joint and two were placed too radially, probably subperiosteally (Figure 3). These three patients remained asymptomatic despite leaving metalwork in situ. Four other patients had screws placed into the radio-carpal joint, all requiring removal of metalwork within two months. Two of these patients had a significant collapse of the fracture and backing out of screws, probably because they were not locked into the plate properly.

One patient sustained an ipsilateral radial head fracture with an Essex-Lopresti lesion, severe pain, stiffness, and poor function despite the removal of metalwork. Six patients had metalwork removed for persistent mild aches with no radiological evidence of complications.

## Discussion

In 1951, Gartland and Werley suggested that manipulation and casting alone produces an unsatisfactory result in 32% of patients, which is unacceptably high [13]. A comparative study by Arora et al. suggested that volar locking plates provide significantly better radiological results than manipulation and casting alone in patients over 70 [9]. However, they suggested that clinical and functional results did not differ between the two groups in the medium term (after a mean of 4.5 years follow-up). They propose that conservative treatment may be the preferred method of treatment in this age group, as these patients had a 0% complication rate (excluding malunion). Early functional results are improved by internal fixation due to faster rehabilitation [6]. Several clinical and biomechanical studies have demonstrated that fixed-angle volar locking plates are an effective means of obtaining and holding reduction of unstable distal radius fractures [2,7,14-16]. This has a particular advantage in osteoporotic bone and the angular stability is sufficient to allow early mobilisation and return to self-care, work and sport [2,6,8].

Fewer studies have looked specifically at variable-angle locking screws or whether their locking mechanisms offer the same strength of construct. Wong et al. published similar results to ours in their prospective cohort study of variable-angle locking plates using a threaded screw head system called Smartlock® [8]. The Aptus® plate uses the Tri-Lock® system whereby cams grip onto the plate by radial bracing. This has proven adequate in holding reduction until fracture healing if the screws are locked into the plate properly. The Tri-Lock® system requires a final twist of the screwdriver to lock the screw into the plate after initial tightening. This final twist needs to be quite forceful to confirm locking with a distinctive click. It is likely that this did not occur with the four screws that backed out in two patients. However, the correct use of a variable-angle device should avoid repeated repositioning of the plate and the associated ‘pepper-potting’ of the already osteoporotic bone with multiple drill holes that may not be used.

As with some other volar locking plates, the Aptus® plate follows an anatomical design with the screw holes facing in pre-determined optimal directions. The Tri-Lock® screws can be angled 15˚ in any direction from each hole’s primary trajectory. However, the locking mechanism will fail if the surgeon is tempted to over-angle the screw in order to catch a fracture fragment. This may have been the cause of failure in two patients in our cohort. Secondary reduction of fractures using the anatomically contoured plate to restore volar tilt and radial inclination is a well-known technique [12]. An additional benefit of the Tri-Lock® variable-angle locking screws seems to be the ability to use the screw to ‘fine-tune’ fracture reduction. Each screw can be used to reduce and compress fracture fragments by using the screwdriver as a joystick, to manoeuvre the fracture fragments to their anatomical position before locking the screw head into the plate. The Tri-Lock® variable-angle locking screws have no threads and therefore the locking mechanism continues to work if the screw is locked at a slightly different angle to the initial drilling. This may offer a significant advantage over fixed-angle distal radius plates in which the screw angle remains predetermined by initial drilling, therefore preventing secondary joystick reduction of comminuted fracture fragments. The absence of threads may also reduce the incidence of cold-welding of screws onto the plate. There were no problems with metalwork removal from patients in our cohort.

Previous studies suggest that it is safe to mobilise wrists immediately after fixation with a volar locking plate which is due to the fact that the threaded locking screws and locking plate can provide an internal-external fixator, which acts as a single unit to support and hold the bone. This is particularly important in osteoporotic bone and in very comminuted fractures [8]. The Aptus® plate has proven a strong enough construct to hold fracture reduction until union, even with immediate mobilisation of the wrist postoperatively. Other studies suggest that the duration of cast immobilisation makes no difference to function either in the short term or the long term [4]. Surgeons at the regional hand unit routinely mobilise their patients out of cast within a week of fixation. At district general hospital, surgeons do not follow any protocols in this regard and appear to be more cautious in mobilising their patients’ wrists. We found a positive correlation between the duration of cast immobilization and loss of radial and ulnar deviation and forearm rotation. Initial severity and classification of fracture did not significantly influence the degree of stiffness in our study. However, we found that immobilising patients’ wrists for four weeks or more post-operatively causes increased stiffness, even at the one-year follow-up.

An 11% rate of misplacing screws is relatively high as compared with other studies. This suggests that it is technically quite difficult to place individual screws accurately, especially in complex, intra-articular fractures. Variable-angle locking mechanisms may add complexity to volar plating, which can lead to screw misplacement or slight loss of reduction intraoperatively. However, our study confirmed that there is no clinical difference between patients who had a ‘good reduction’ as per Wong’s criteria or a less good one. Complication rates of fixed-angle volar locking plates reported in the literature are relatively high and comparable to our results in several case series [5,9-10]. The overall percentage of complications in our study was 18% in comparison to a range of 22% to 48% with a mean of 35% in other studies checked. Wong et al. reported only two complications in 35 patients treated with the Smartlock® variable-angle locking system [8]. This may suggest that variable-angle devices fail less frequently than fixed-angle devices, possibly because the raft of screws can be customised to the curve of the subchondral bone in each patient.

Although the positions of all fractures were improved with surgery, 46% of patients’ wrists did not achieve ‘good reduction’ as per Wong’s criteria. This limitation of poor radiological results may be due to pitfalls in using non-standardised electronic systems to measure radiographic findings, or it may demonstrate a learning curve of 18 different surgeons’ first experiences with a new device, or it possibly reflects the complexity of the fractures being fixed [14]. Another limitation to our study was that nine patients had incomplete follow-up and four were excluded from the study as they were lost to follow-up. It is possible that any postoperative complications for these patients were diagnosed and treated at other facilities.

## Conclusions

Relatively high complication rates using volar locking plates may add weight to the arguments for the nonoperative management of distal radius fractures. However, despite some malunions and complications, the Aptus® plate seems to provide good clinical and functional outcomes at one year. Benefits of this system are the ability to target specific fracture fragments and to fine-tune reduction using the screwdriver as a joystick. Patients can be mobilised immediately postoperatively without losing fracture reduction allowing for earlier return to work, hobbies and self-care. However, we acknowledge the need for extra caution with individual screw placement to avoid breaching the joint.
